# Non invasive in vivo investigation of hepatobiliary structure and function in STII medaka (*Oryzias latipes*): methodology and applications

**DOI:** 10.1186/1476-5926-7-7

**Published:** 2008-10-06

**Authors:** Ron C Hardman, Seth W Kullman, David E Hinton

**Affiliations:** 1 Duke University, Environmental Sciences and Policy Division, Nicholas School of the Environment and Earth Sciences, LSRC A333, Durham NC, USA

## Abstract

**Background:**

A novel transparent stock of medaka (*Oryzias latipes*; STII), recessive for all pigments found in chromatophores, permits transcutaneous imaging of internal organs and tissues in living individuals. Findings presented describe the development of methodologies for non invasive in vivo investigation in STII medaka, and the successful application of these methodologies to in vivo study of hepatobiliary structure, function, and xenobiotic response, in both 2 and 3 dimensions.

**Results:**

Using brightfield, and widefield and confocal fluorescence microscopy, coupled with the in vivo application of fluorescent probes, structural and functional features of the hepatobiliary system, and xenobiotic induced toxicity, were imaged at the cellular level, with high resolution (< 1 μm), in living individuals. The findings presented demonstrate; (1) phenotypic response to xenobiotic exposure can be investigated/imaged in vivo with high resolution (< 1 μm), (2) hepatobiliary transport of solutes from blood to bile can be qualitatively and quantitatively studied/imaged in vivo, (3) hepatobiliary architecture in this lower vertebrate liver can be studied in 3 dimensions, and (4) non invasive in vivo imaging/description of hepatobiliary development in this model can be investigated.

**Conclusion:**

The non-invasive in vivo methodologies described are a unique means by which to investigate biological structure, function and xenobiotic response with high resolution in STII medaka. In vivo methodologies also provide the future opportunity to integrate molecular mechanisms (*e.g*., genomic, proteomic) of disease and toxicity with phenotypic changes at the cellular and system levels of biological organization. While our focus has been the hepatobiliary system, other organ systems are equally amenable to in vivo study, and we consider the potential for discovery, within the context of in vivo investigation in STII medaka, as significant.

## Background

The majority of our understanding of vertebrate hepatobiliary disease and toxicity has been derived from mammalian liver studies [1-5]. We know comparatively less about piscine biliary disease and toxicity, though we are beginning to gain greater insight into piscine hepatobiliary structure/function relationships [6-17]. Because our understanding of the piscine biliary system has lagged, particularly in a comparative sense, our ability to interpret and communicate biliary disease and toxicity in aquatic species has remained limited. By example, cholestasis (impaired and/or inhibited bile transport) has never been described in fish, a fact more representative of our lack of understanding/investigation, as opposed to the lack of occurrence of this physiological response.

Because the vertebrate liver is a common target organ of toxicity, largely due to the emergence and prevalence of modern xenobiotics (*e.g*., environmental contaminants, pharmaceuticals) in society over the last century [18-24], and the fact that various vertebrate systems (fish, rodent, avian, primate, human) are applied to understanding the mechanisms and modes of actions involved in xenobiotic induced injury, a betterment of our comparative understanding, and ability to investigate and interpret hepatotoxicity in the piscine hepatobiliary system, is essential. By example; the presence of personal care products and pharmaceuticals (PCPPs) [25-32], persistent environmental contaminants (POPs) [21,22,33-35], and the widespread application of antibacterial agents, pesticides, and hormones in aquaculture [36-39] present ecotoxicological and, because of the human consumption of fish, human health related concerns, that are likely to persist for decades. These and other environmental contaminants necessitate enhancement of our ability to understand hepatobiliary disease and toxicity in piscine species.

Advancement of our understanding of the piscine hepatobiliary system is not relegated to environmental concerns alone. Because of their many advantages (*e.g*., small size, relative ease of care and use, potentially higher statistical power due to large study cohorts), small fish animal models such as medaka, zebrafish, and fathead minnow have seen increasing application in biomedical research (*e.g*., carcinogenesis, mutagenesis, functional genomics, toxicogenomics) [40-45].

The see-through medaka (*Oryzias latipes*; STII), recessive for all pigment genes of chromatophores (iridophores, melanophores, xanthophores and leucophores) [46], is a unique small fish animal model that enables high resolution (< 1 μm) in vivo imaging of biological structure and function at virtually all levels of organization, from subcellular to gross anatomical [12,47]. Exhibiting no expression of leucophores and melanophores, and minimal expression of xanthophores and iridophores, STII medaka are essentially transparent throughout their life cycle. In embryo, larval and juvenile STII medaka (from 3 to 60 days post fertilization (dpf)) it is possible to image internal cells, tissues and organs, and generate three-dimensional (3D) reconstructions from in vivo imaging. We present here an overview of our findings from in vivo investigations in STII medaka that demonstrate the utility of this experimental in vivo system. Specifically, we show that: phenotypic response to xenobiotic exposure can be investigated/imaged in vivo with high resolution (< 1 μm); hepatobiliary transport of solutes from blood (sinusoid) to bile (intrahepatic biliary passageways) can be qualitatively and quantitatively studied in vivo; hepatobiliary development in this model can be described/investigated via non invasive in vivo observations; and hepatobiliary structure/function in this lower vertebrate liver can be studied in 3D.

Our purpose here is to share in vivo methodologies and present examples of applications of these methodologies to in vivo investigation. First we describe the in vivo methodologies developed, and then give specific examples of the successful application of these methodologies to the evaluation of hepatobiliary structure, function, development, and xenobiotic response, in both 2D and 3D. Because much of our recent research has focused on the piscine hepatobiliary system, namely biliary system toxicity, this organ system will be emphasized in the examples provided.

## Methods

### Medaka

For decades color mutant strains of medaka (*Oryzias latipes*), acquired from natural and commercially available populations, have been maintained in the Laboratory of Freshwater Fish Stocks at Nagoya University, Japan. Cross breeding from these stocks was used to produce a stable "transparent" strain of medaka [46], from which our STII medaka colony, maintained at Duke University since 2002, was derived. Fish care and maintenance was provided daily in accordance with Duke University IACUC approved animal protocols (A117-07-04; A141-06-04; A173-03-05). Brood stock were housed in a charcoal filtrated and UV treated recirculating system (City of Durham, NC water) maintained at 25 +/- 5°C. Water chemistries were maintained at; pH (7.0–7.4), dissolved oxygen (6–7 ppm), ammonia (0–0.5 ppm), nitrite (0–0.5 ppm) and nitrate (0–10 ppm). Brood stock were maintained on a diel cycle of 16:8 hr light:dark. Unconsumed diet, detritus and associated algal material were removed from brood stock tanks daily. Eggs and egg clusters, collected daily, were separated, cleaned in embryo rearing medium (ERM), and individual fertilized eggs maintained in ERM at 25°C for stock maintenance. Medaka larvae were fed a commercial ration of ground (100 μm) Otohime β daily (Ashby Aquatics, West Chester, PA). In addition, all brood stock fish diets were supplemented with *Artemia nauplia *(hatched brine shrimp) at least once per day, seven days per week.

### Microscopy

Widefield microscopy was performed on a Zeiss Axioskopp equipped with DAPI/TRITC/FITC fluorescence filter cube set (DAPI/UV: Ex 360–380 nm/Em All Vis > 400 nm, FITC: Ex 450–490 nm/Em 515–565 nm, TRITC: Ex 528–552 nm/Em 578–632 nm), Zeiss Plan Neofluar 5×/0.15, 10×/0.3, 20×/0.5, 40×/0.85 pol, and 100×/1.3 oil objectives, Photometrics CoolSnap digital imaging system (2048 × 2048-element array) and IP Lab (V. 3.0) image acquisition software (Scanalytics). A xenon lamp was used for excitation. For confocal fluorescence microscopy a Zeiss 510 Meta system with Zeiss LSM 5 Axiovision image acquisition software, Argon and HeNe laser, and Carl Zeiss C-apochromatic 40×/1.2 and C-apochromatic 10×/0.45 objectives, was used. For brightfield microscopy a Nikon Eclipse E600 with a Nikon DXM 1200 digital capture system, halogen light source, Nikon plan neo-fluor 10×/0.3 wd16, plan neo-fluor 20×/0.5 wd2.1, plan neo-fluor 40×/0.75 wd0.72, and plan apo 60×/1.4 wd0.21 (oil) objectives, was used. A Nikon SZM 1500 with a Nikon DXM 1200 digital capture system, Nikon HR plan apo 1× WD54 and Nikon HR 0.5× WD136 objectives was also employed for brightfield dissecting microscopy. All transmission electron microscopy (TEM) was performed at the Laboratory for Advanced Electron and Light Optical Methods (LAELOM), College of Veterinary Medicine, North Carolina State University, on an FEI/Philips EM 208S Transmission Electron Microscope.

### Software

Image analysis and compilation was performed with EclipseNet (Nikon, USA), Adobe Photoshop (Adobe, Inc.), ImageJ (NIH, V1.32j), IP Lab software (Scanalytics, Inc., version 3.55), and Zeiss Image Browser (Carl Zeiss). All 3 dimensional reconstructions and analyses were performed with Amira 3D (Mercury Computer Systems, Berlin).

### Chemicals and fluorescent probes

A list of fluorescent probes is provided in Table 1. The primary fluorescent probes employed were; 7-benzyloxyresorufin, β-Bodipy C5-HPC, DAPI {4',6-diamidino-2-phenylindole, dihydrochloride}, and fluorescein isothiocyanate (FITC). All fluorescent probes were administered to STII medaka via aqueous bath in concentration ranges listed in Table 1. Duration of exposure times (aqueous bath exposures) varied for each fluorescent probe, and can be derived from the values given in initial and peak fluorescence column. Other chemicals employed: Diethylnitrosamine (N-nitrosodiethylamine, Sigma, N0756), α-napthylisothiocyanate (Sigma, N4525), β-napthoflavone (Sigma, N-3633), tricaine-methane sulfonate (Sigma, E10521), dimethyl sulfoxide (DMSO, Sigma, 276855), Pronase (streptococcal protease, Sigma), Hank's balanced salt solution (Sigma, H5899), and phosphate buffered saline (PBS, sigma).

**Table 1:** Fluorescent probe specifications

**Fluorophore**	**Soluble**	**Ex**	**Em**	**Initial/Peak Assimilation Time (min)**	**Exposure Concentration**
**7-benzyloxyresorufin**	DMSO	560	590	30/45	50 μM
In vivo CYP3A activity. Uptake via gill, in vivo metabolism in gill and gut, good for investigating blood to bile transport, IHBPs, EHBPs and intestinal lumen.					

**7-Ethoxyresorufin**	DMSO			30/45	10–50 μM
In vivo imaging of CYP1A activity. Uptake via gill, in vivo metabolism in gill and liver. CYP 1A2, 2E Substrate					

**Acridine Orange**	H_2_O	500	526		1–5 μM
In vivo labeling of DNA, RNA. Good for apoptosis, interacts with DNA and RNA by intercalation or electrostatic attractions.					

**BODIPY 505/515: 4,4-difluoro-1,3,5,7-tetramethyl-4-bora-3a,4a-diaza-s-indacene**	DMSO	502	510	15/20	5 μM-100 nM
Uptake via gill, active transport through hepatobiliary system, concentrative blood to bile transport, and secretion from gall bladder into gut lumen. Good for elucidation of gill, IHBP, EHBP, intestinal lumen. Non-Polar, Lipophilic.					

**BODIPY FL C5-ceramide: N-(4,4-difluoro-5,7-dimethyl-4-bora-3a,4a-diaza-s-indacene-3-pentanoyl)sphingosine**	DMSO	358	461	20/45	5 μM–100 nM
Putative passive transport in vivo. Uptake via gill, transport through cardiovascular and hepatobiliary systems and secretion from gall bladder into gut lumen.					

**Bodipy Verapamil**	DMSO	504	511	20/60	
In vivo Bodipy verapamil localized to hepatocytic cytosol in discrete vesicles. Transport to bile was not observed in the time frame assayed, which was 90 minutes.					

**BODIPY^® ^493/503: 4,4-difluoro-1,3,5,7,8-pentamethyl-4-bora-3a,4a-diaza-s-indacene (BODIPY^® ^493/503)**	DMSO	493	504	15/20	10 μM
Uptake via gill, active transport through hepatobiliary system, concentrative blood to bile transport, and secretion from gall bladder into gut lumen. Good for elucidation of gill, IHBPs, EHBPs, intestinal lumen. Lipophilic, amphiphilic.					

**BODIPY^® ^581/591 C5-HPC (Phosphocholine) PC: 2-(4,4-difluoro-5,7-dimethyl-4-bora-3a,4a-diaza-s-indacene-3-pentanoyl)-1-hexadecanoyl-sn-glycero-3-phosphocholine**	DMSO	582	593	15/20	30 nM
OIn vivo labeling of intrahepatic and extrahepatic biliary system. Uptake via gill. Hepatobiliary transport to gut lumen. Diffuse fluorescence in hepatocyte cytosol.					

CellTrace™ Oregon Green^® ^488 carboxylic acid diacetate, succinimidyl ester (carboxy-DFFDA, SE) *cell permeant* *mixed isomers	DMSO	<300	none	15/20	10–100 μM
In vivo labeling of hepatic nuclei, potential for detection of apoptosis					

**DAPI: 4',6-diamidino-2-phenylindole, dihydrochloride**	H2O, DMF	358	461	15/60	0.3 – 3.0 μM
In vivo nuclear labeling of virtually all cell types associated with gill, gut, liver, and cardiovascular system.					

**DAPI Diacetate: 4',6-diamidino-2-phenylindole, diacetate**	H2O, MeOH	358	461	15/60	0.3 – 3.0 μM
DAPI diacetate is water-soluble form. In vivo nuclear labeling.					

**Fluorescein-5-isothiocyanate (FITC 'Isomer I')**	DMSO	494	519	10/30	1 μM – 50 nM
Excellent in vivo probe for elucidating biliary system. In vivo labeling of intrahepatic and extrahepatic biliary system. Hepatobiliary transport to intestinal lumen. Uptake via gill.					

**MitoTracker^® ^Green FM**	DMSO	490	516	20/30	25–200 nM
In vivo labeling of hepatocytes.					

**SYTO^® ^16 green fluorescent nucleic acid stain**	DMSO	488	518	15/30	10 nM-1 μM
In vivo nuclear labeling of epithelia and endothelia, putative in vivo probe for apoptosis.					

**SYTO^® ^27 green fluorescent nucleic acid stain**	DMSO	495	537	15/50	10 nM-1 μM
Apoptosis.					

**SYTOX^® ^Orange nucleic acid stain**	DMSO	547	570	15/50	0.1–5 μM
Apoptosis					

**YO-PRO^®^-1 iodide (491/509)**	DMSO		509	15/60	1 μM
Apoptosis					

**YOYO-1 Iodide (491/509)**	DMSO	491	509	15/50	2 – 5 nM
In vivo accumulated in interstitial spaces, some labeling of vasculature. Typically used for assays for cell enumeration, cell proliferation and cell cycle.					

Eighteen fluorescent probes were selected for in vivo application based on their molecular weight, utility in elucidating desired structure/function, biocompatibility, and in vivo transport properties. The fluorescent probe, a description of application(s), the molecular weight (M.W.), solvent used for stock preparation (solubility), fluorescence excitation and emission wavelengths (Ex/Em), initial and peak fluorescence times (Initial/Peak Fluorescence (min)), and effective aqueous exposure concentrations are given.

### Xenobiotic exposures

Reference hepatotoxicants α-napthylisothiocyanate (ANIT) and diethylnitrosamine (DEN) were used for comparative study of responses of the hepatobiliary system. ANIT is a well described biliary toxicant in rodent models that induces hallmark responses in the mammalian liver, namely: cytotoxicity in biliary epithelium of bile ductules and ducts, cholestasis [48-51], and biliary tree arborization (biliary epithelial cell hyperplasia) [52-54]. DEN, a complete carcinogen, is a widely employed reference hepatotoxicant that has been used in fish [55].

ANIT Exposures: Cohorts (10 to 30 fish) of STII medaka were exposed to aqueous ANIT to target hepatocytes and biliary epithelia for toxic response. Exposures were in aqueous bath during various stages of development, under acute and chronic exposure conditions. Controls consisted of untreated medaka, and those that received DMSO (ANIT solvent). Studies were designed to evaluate hepatobiliary structure/function during the onset, progression, and recovery from ANIT induced changes. All exposures were done in 750 ml wide-bottom glass rearing beakers at 25°C, 16 hr light/8 hr dark cycle. Aqueous bath exposure medium was 1:3, ERM:de-ionized water. Acute exposures: medaka were subjected to single exposures of ANIT at concentrations from 0.25 μM to 10 μM ANIT and assessed at multiple time points (*e.g*., 5 minutes, 15 minutes...6, 12, 24, 48, 72 and 96 hrs, and at day 10, 30 and 60 post exposure). Chronic exposures: medaka were re-exposed every 3 days, or once weekly (static renewal baths), and examined at the same points given for acute exposures. DEN exposure was for 48 hrs at 200 ppm in aqueous bath, after which time medaka were reared for 10 months under normal conditions. At given time points during ANIT and DEN exposure regimes, subpopulations of medaka were removed from an exposure cohort for in vivo investigations, histological, immunohistochemical, and transmission electron microscopy (TEM) studies.

### In vivo methodologies

As an overview, in vivo investigation in STII medaka can be considered to be comprised of three primary components, which describe a "system" of study; (1) sedation, (2) microscopy/imaging technologies, and (3) fluorescent probes.

To facilitate in vivo imaging of larval and older STII medaka (> 8 dpf) non lethal sedation was necessary, particularly for cellular level investigations. Optimal sedation was achieved with 10 μM tricaine methane sulfonate (MS-222). STII medaka were placed in a solution of 10 μM MS-222 (ERM as solvent), as soon as medaka were anaesthetized (immotile) they were removed from the sedative bath and placed in fresh ERM:deionized water (1:3). Sedated medaka were then placed on a glass depression well slide with enough ERM:deionized water to fill the depression well (~400 μl), oriented in a preferred anatomical position, and the depression well slide was sealed with a cover-slip. For optimal image clarity medaka were positioned so the dermis was in contact with the coverslip, which minimized optical distortion that may result from image diffraction through the fluid space between dermis and coverslip. Once mounted on a depression well glass slide medaka were imaged live with brightfield, and widefield and laser scanning confocal fluorescence microscopy (LSCM). Each was found to offer specific advantages, which will be illustrated through the in vivo findings presented. A brief overview of considerations for each imaging modality follows.

**Figure 1:**
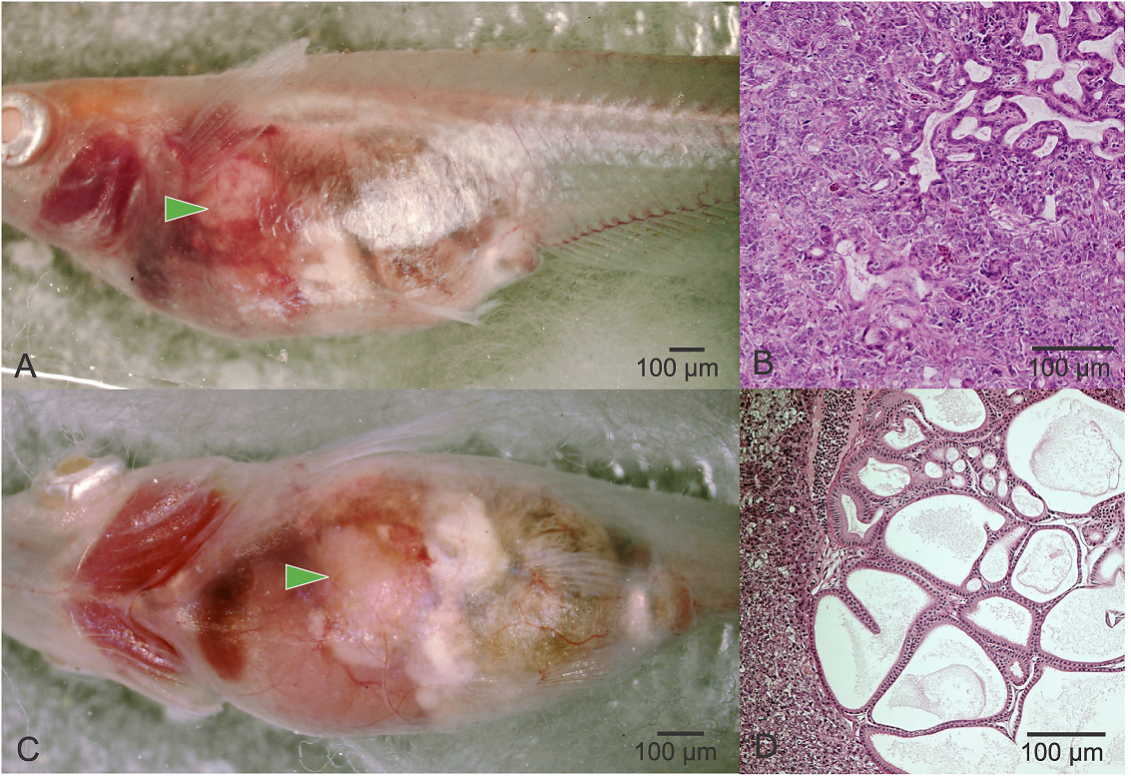
**In vivo imaging of tumor formation in STII medaka: brightfield microscopy**. Neoplastic response following early life stage exposure of STII medaka to the reference hepatocarcinogen diethylnitrosamine (DEN). Medaka acutely exposed at early life stages to DEN were followed serially, and at 10 months hepatic tumors were imaged through the abdominal wall. (A and C) In vivo imaging (brightfield) of hepatic tumor formation (green arrowheads) in DEN exposed medaka, showing enlargement of total liver mass and altered vasculature. Histopathological assessment of the tumor showed mixed hepatocellular (B) and cholangiocellular carcinomas (D). Biliary hyperplasia (D) was characterized by a single layer of biliary epithelium lining large cystic spaces in the liver. Opaque white tissue in brightfield images (A&C) is ovary, whereas the gut occupies caudal most region of the abdominal cavity.

**Figure 2:**
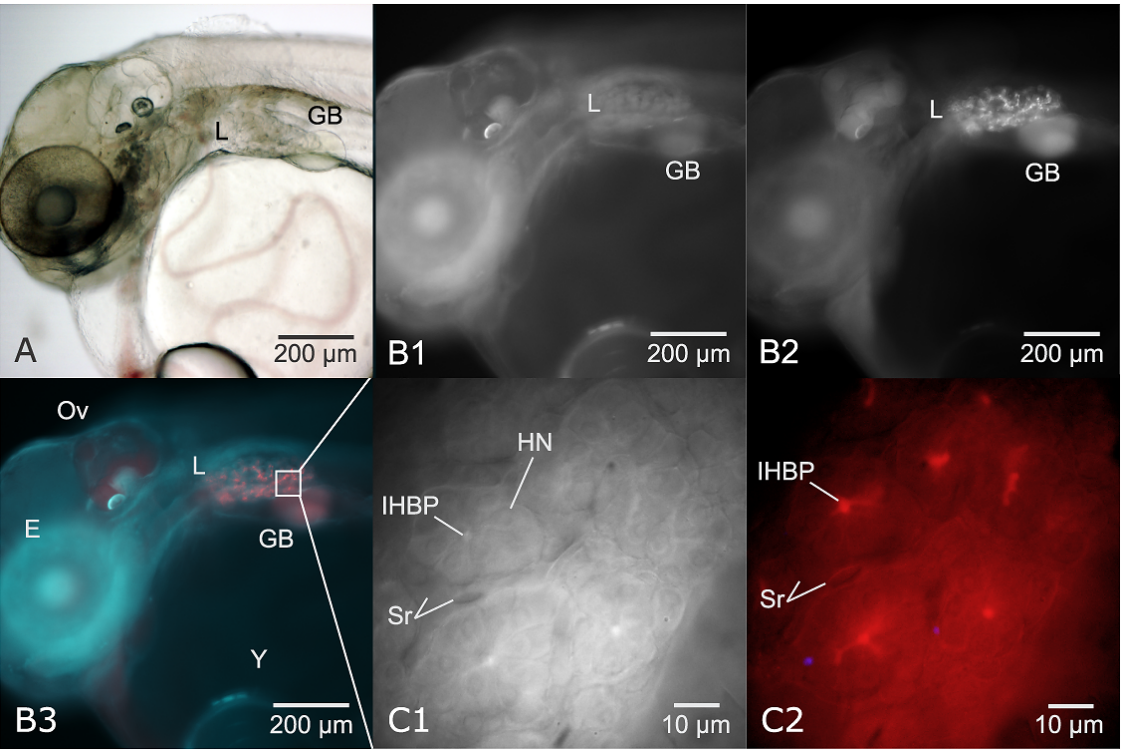
**In vivo imaging of hepatobiliary metabolism, transport, and hepatic morphology**. Utilizing endogenous tissue autofluorescence in tandem with exogenous fluorescent probes allowed in vivo elucidation of biological structure and function. Illustrated is the in vivo application of 7-benzyloxyresorufin (7BR) for detection of CYP3A metabolic activity. Dechorionated embryos exposed (aqueous bath) to the CYP3A substrate 7BR exhibited 7-benzyloxyresorufin-O-dealkylation (BROD) activity, which resulted in the generation of the fluorescent metabolite resorufin (red). All in vivo images from an individual STII medaka, 6 dpf. (A) Brightfield microscopy, showing liver (L) and gall bladder (GB). (B1) Same animal as in frame A, imaged with widefield fluorescence microscopy (DAPI/UV excitation) illustrating tissue autofluorescence. (B2) Widefield fluorescence (TRITC) image capture of resorufin (indicative of CYP3A metabolic activity) fluorescence in the intrahepatic biliary passageways of the embryonic liver. Resorufin fluorescence is distinct and limited to the intrahepatic biliary passageways of the liver (L) and gall bladder (GB). (B3) Color composite of B1 and B2, DAPI/TRITC image captures, illustrating resorufin fluorescence (red) in the liver (L) and gall bladder (GB). (C1 and C2) In vivo imaging of region of interest in frame B3 (white square) showing in vivo phenotype of hepatic parenchyma at 6 dpf. Six to 8 hepatocytes were observed (in transverse section) to surround a central bile passageway (IHBP) at their apical membranes. C2 illustrates concentrative transport of resorufin from hepatocellular cytosol to intrahepatic biliary passageways (IHBPs), indicated by increased fluorescence in the tubule lumen, or intrahepatic biliary passageway. Red blood cells were observed actively circulating through hepatic sinusoids (S/r). Hepatocyte nuclei (HN).

**Figure 3:**
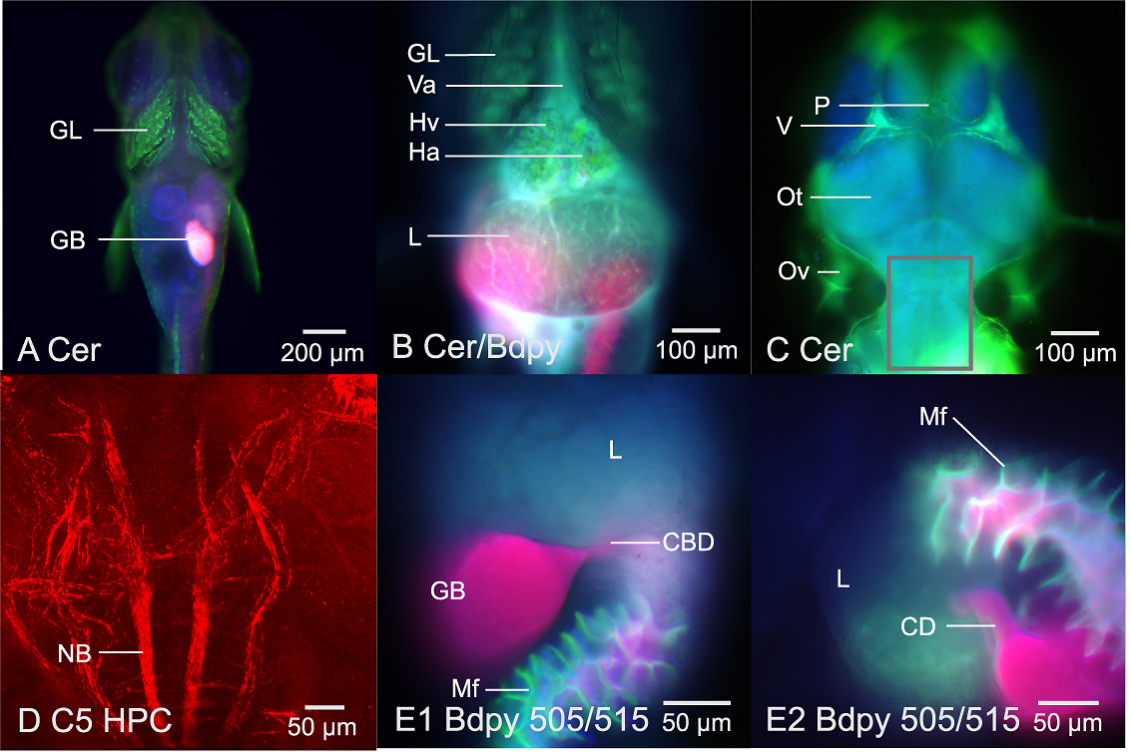
**Differential uptake and transport of fluorescent probes β-Bodipy C5-HPC, Bodipy FL C5 Ceramide and Bodipy 505/515: Widefield and confocal fluorescence microscopy**. (A – C) β-Bodipy C5 ceramide uptake and distribution: the ceramide fluorophore (green) exhibited properties consistent with passive diffusion across cell membranes, with distinct uptake over the gill (GL) and transport through the cardiovascular system. The fluorophore was not observed to cross the blood-brain barrier (C), though it persisted in vasculature, with a residence time of hours to days (depending on exposure regime). (D) In contrast, β-Bodipy C5 phosphocholine (HPC) was observed to label neurons in the hind brain of STII medaka. Frame D is a confocal fluorescence image (from 3D projection) of β-Bodipy C5 phosphocholine labeling neural bundles in the corpus cerebelli, crista cerebellaris and medulla, 90 minutes post fluorophore exposure (aqueous bath) (STII medaka, 18 dpf). Corpus cerebelli, crista cerebellaris and medulla are region of interest indicated by gray rectangle in frame (C). (E1 and E2) Widefield fluorescence microscopy of Bodipy 505/515 secretion (red fluorescence) from gall bladder (GB) through the cystic duct (CD) and common bile duct (CBD) into the gut lumen. Mucosol folds of the gut (Mf) are the result of β-Bodipy C5 ceramide fluorescence (co-administered with Bodipy 505/515). Ventral aorta (Va), Heart Ventricle (Hv), Heart Atrium (Ha), Liver (L), Pineal Gland (P), Optic Tectum (Ot), Otic Vesicle (Ov), Vasculature (V).

**Figure 4:**
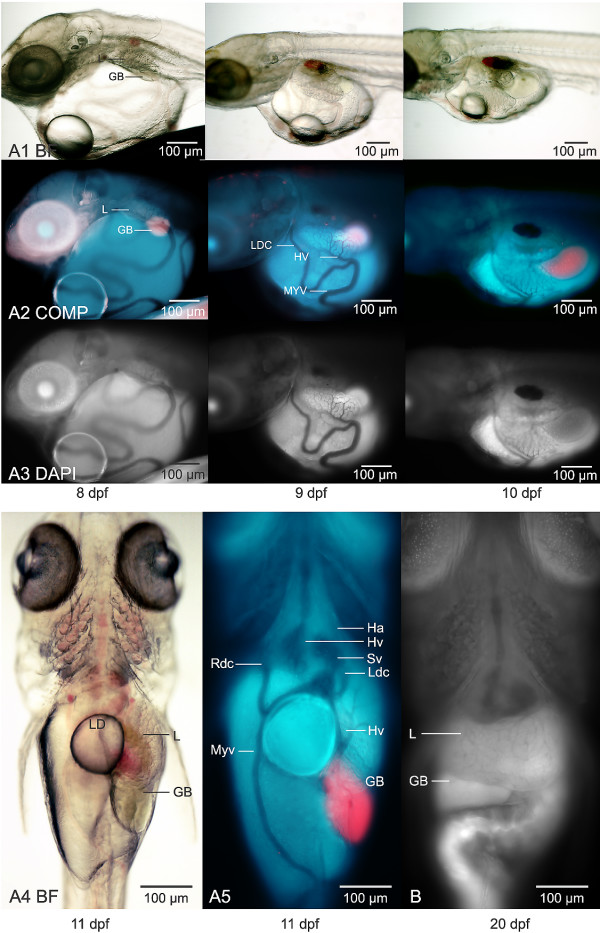
**In vivo investigations of hepatobiliary development**. (A1 – 1^st ^row) Brightfield microscopy. (A2-2^nd ^row) Composites of DAPI and TRITC image captures, revealing gall bladder (GB) position (red). (A3 – 3^rd ^row) DAPI autofluorescence elucidating vasculature. At day of hatching, 8 dpf, the liver was found as the left lateral longitudinal liver leaflet (L5) phenotype, left lateral and ventral to the 3rd somite (A1 – A3, 1^st ^column). From 8 to 11 dpf the L5 liver and gall bladder descended to the ventral abdomen, with marked restructuring of the visceral and hepatic vasculature. Translocation of the L5 liver and gall bladder to the ventral abdomen (A4, A5) was characterized by: descent of the liver and GB, which remained in a longitudinal position, yolk resorption, the disappearance of the stomatodeal and proctodeal membranes (not shown), the onset of peristalsis, and the beginning of respiration. (A4) Brightfield microscopy, 11 dpf, ventral view, showing liver, gall bladder and lipid droplet. (A5) Widefield fluorescence microscopy, DAPI/TRITC composite, ventral view, elucidating liver, gall bladder and vasculature. The onset of metamorphosis of the hepatobiliary system to a transverse position in the rostral abdominal cavity (adult phenotype) began at 11 dpf. (B) Widefield fluorescence microscopy, DAPI image capture, ventral view. By 20 dpf the liver and gall bladder were transverse in the ventro-rostral abdominal cavity, marking the attainment of the adult phenotype. As can be discerned from the images shown, marked restructuring of the vasculature accompanied metamorphosis of the liver and gall bladder from an embryonic to adult phenotype. While such observations are purely descriptive, they permitted characterization of hallmark events in hepatobiliary development and helped establish normalcy in vivo. Liver (L), Gall Bladder (GB), Heart Atrium (Ha), Heart Ventricle (Hv), Sinus Venosus (Sv), Hepatic Vein (Hv), Left Duct of Cuvier (Ldc), Median Yolk Vein (Myv), Lipid Droplet (LD).

**Figure 5:**
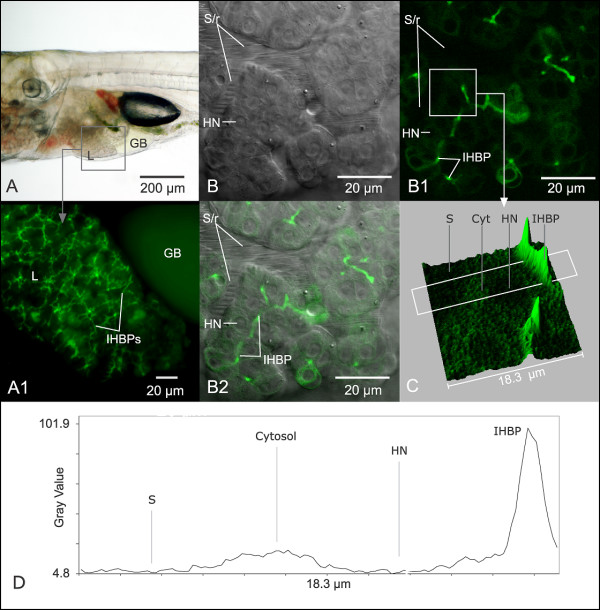
**In vivo imaging of hepatobiliary transport**. Fluorophores such as β-Bodipy C5 phosphocholine, shown here, enabled in vivo elucidation of the biliary system and quantitation of blood to bile transport. (A) Brightfield microscopy of STII medaka at 30 dpf. Green algae can be seen in transport through lumen of the gut. (A1) Widefield fluorescence microscopy of region of interest indicated by gray square in A, showing β-Bodipy C5 phosphocholine fluorescence in intrahepatic biliary passageways (IHBPs) of the liver (L) and gall bladder (GB). (B) Confocal DIC microscopy, STII medaka, 9 dpf: Clearly resolved were hepatocytes and their nuclei/nucleoli. In longitudinal section 2 rows of hepatocytes characterize parenchymal architecture. Stacked ovate structures are red blood cells in circulation through the sinusoids (S/r). Red cells appear flattened due to active circulation of cells through sinusoids, and resulting distortion during imaging. (B1) Same as B: Single frame from in vivo confocal image stack capturing β-Bodipy C5 phosphocholine (green fluorescence) in transport from blood to bile, through intrahepatic biliary passageways (IHBPs) of the liver (imaged in vivo 30 minutes post fluorophore administration). (B2) Composite of frames B and B1 localizing fluorophore transport to area between apical membranes of adjacent hepatocytes, suggesting concentrative transport of the fluorophore into IHBPs. (C and D) Frame C is a surface map of region of interest (white square) in frame B1, illustrating concentrative transport of the fluorophore from sinusoidal space (S) to bile space (IHBP). (D) Quantitative evaluation of the white rectangular region of interest in frame B1, spanning an 18.3 μm area from blood to bile (sinusoid to canaliculus), suggested β-Bodipy C5 phosphocholine concentration (fluorescence intensity) to be ~20 times greater in the canalicular (IHBP) vs. sinusoidal spaces (S). Also evident is an increase in cytosolic concentration of the fluorophore, while no fluorescence was detected in the nucleus. These types of studies demonstrated concentrative transport of fluorescent probes from blood to bile can be imaged and quantitatively assessed in vivo. Confocal images captured with C-apochromatic objective, 1.2 NA w/correction, 488 nm excitation, Zeiss LSM 510. Field size: 76.8 × 76.8 μm.

**Figure 6:**
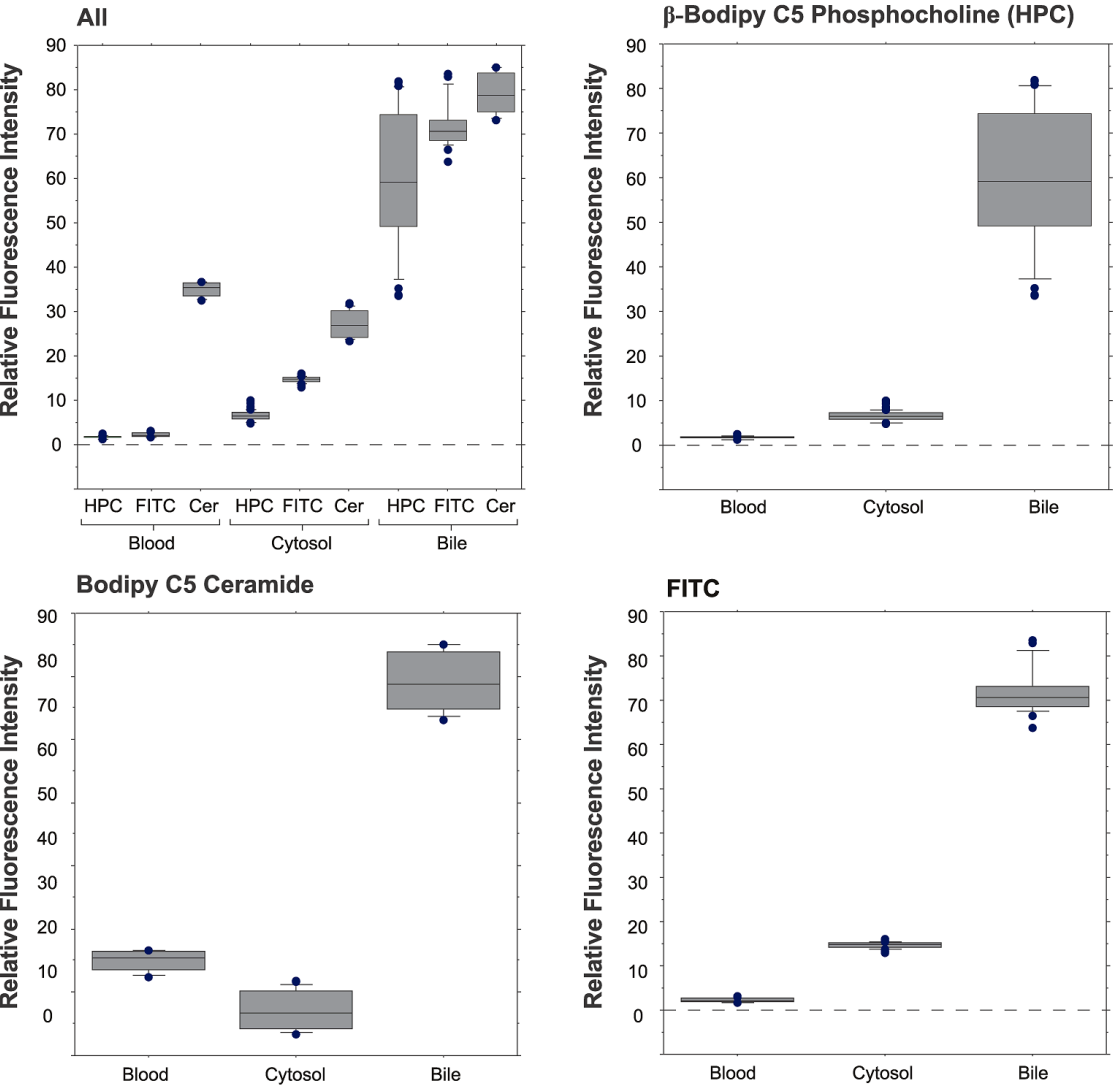
**Quantitative analysis of blood to bile transport in vivo (STII medaka, 12 dpf): β-Bodipy C5 Phosphocholine (HPC), Fluorescein Isothiocyanate (FTIC) and Bodipy C5 ceramide**. Box plots (area averages) and statistical indices: Quantitative analysis of differential blood to bile transport between β-Bodipy C5 phosphocholine (HPC), fluorescein isothiocyanate (FTIC) and Bodipy C5 ceramide. Fluorophores were imaged in vivo at peak uptake times (60 minutes for C5 ceramide, 45 minutes for both FITC and HPC). Differences in blood to bile transport between all three fluorophores were suggested when measured values (means) of fluorescence intensity across sinusoid, cytosol and canalicular spaces were evaluated. The most marked differences were between β-Bodipy C5 phosphocholine and fluorescein isothiocyanate, and Bodipy C5 ceramide. For instance: Where ceramide and FITC showed no statistical difference in concentration in the canaliculus, there were marked differences in concentration, and temporal variation, in cytosol and sinusoid, suggesting differences in transport kinetics.

**Figure 7:**
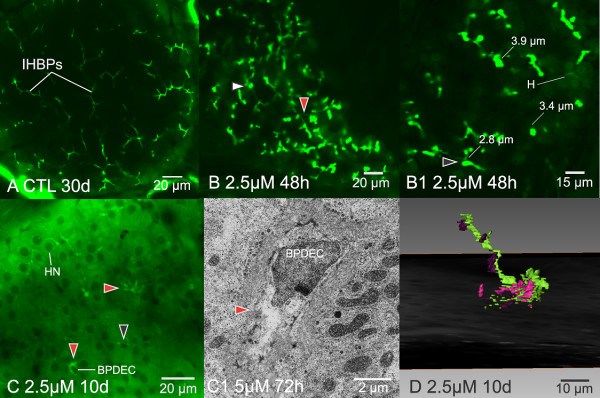
**In vivo imaging of hepatobiliary toxicity: canalicular attenuation/dilation and bile preductular lesions**. (A) In vivo confocal image of untreated medaka liver (30 dpf), illustrating normal appearance of IHBPs, characterized by uniform diameter. (B – B1) In vivo confocal image, single optical section, of ANIT treated medaka liver (24 dpf) illustrating appearance of dilated and attenuated bile canaliculi (red arrowhead points to attenuation, white to dilation), 48 hrs post exposure to 2.5 μM ANIT. Canaliculi were elucidated with fluorescein isothiocyanate. Only the intrahepatic biliary passageways are fluorescent (green). Parenchyma is largely non fluorescent, aside from weak and diffuse fluorescence of hepatocellular cytosol. Canalicular dilation/attenuation appeared to be a canalicular constriction/dilation regulatory problem, as no clear alteration to hepatocyte morphology was observed in association with this change. (B1) Dilated canaliculi (gray arrowhead) were found to be up to approximately 3 times normal diameter (*e.g*., 3.9 μm diameter in dilated *vs*. 1.3 μm average diameter in normal canaliculi). Attenuated canaliculi were distinct, appearing as fine sinuous passageways measuring 0.4 μm to 0.8 μm in diameter. (C) Non invasive in vivo confocal image 10 days post exposure to 2.5 μM ANIT (chronic exposure) showing bile preductular lesions (red arrowhead), characterized by loss of preductule membrane integrity and loss of uniformity in preductule lumen diameter. Intrahepatic biliary passageways elucidated here with Bodipy C5 Ceramide. Black arrowhead illustrates normal appearance of bile preductule. (C1) Transmission electron micrograph illustrating changes to bile preductular epithelium (BPDEC) associated with preductular lesions, which showed increased cytosolic area and vacuolation (red arrowhead). In vivo observations helped lead to the hypothesis that ANIT induced BPDEC toxicity is responsible for bile preductular lesions observed, and that these cells are early targets of ANIT. (D) Example of a 3D reconstruction of damaged preductule that revealed the damaged bile passageway (green) was blind ending, not interconnected with other segments of the intrahepatic biliary network (atypical). Also shown are bile preductular epithelial cells (purple), illustrating the foci of alteration was a canaliculo-preductular junction. In (A), (B), and (B1), IHBPs elucidated with FITC, in (C) with Bodipy C5 ceramide.

**Figure 8:**
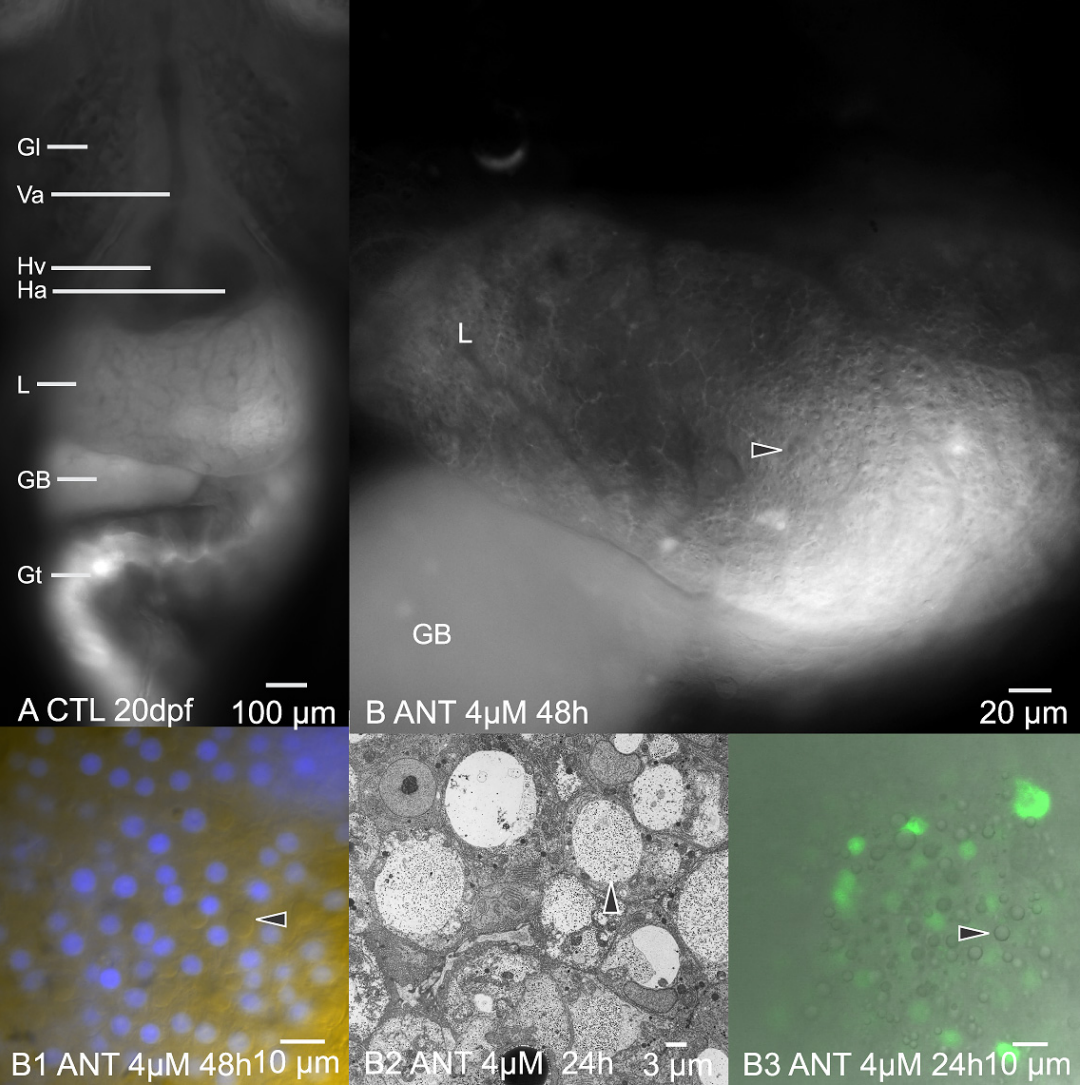
**In vivo imaging of hepatobiliary toxicity: hydropic vacuolation**. Acute exposures to 2 to 6 μM ANIT resulted in a marked "pebbling" phenotype. This terminology was adopted due to the morphological appearance of the liver (L), first observed in vivo with widefield fluorescence microscopy. (A) STII medaka control, 20 dpf, showing the normal smooth appearance of the hepatic parenchyma in vivo, as viewed with widefield fluorescence microscopy. (B) Widefield fluorescence microscopy, FITC image capture, STII medaka, 20 dpf. Shown is the "pebbled" appearance (black arrowhead) of the liver (L) in vivo; distinct at 2 μM to 6 μM aqueous ANIT. This phenotypic response was characterized (in vivo) by ovate structures within the cytosol of hepatocytes, which resulted in a pebbled appearance in the plane of focus in the liver. This phenotype was observed with the aid of autofluorescence alone, no fluorophores were necessary for visualizing this cellular response. (B1) ANIT exposed medaka exhibiting the pebbling phenotype were treated with the nuclear stain DAPI (aqueous bath) to label hepatocyte and biliary epithelial cell nuclei. After 1 hr of DAPI exposure the hepatobiliary systems of medaka were imaged in vivo via widefield fluorescence microscopy. These investigations found that intracellular ovate structures (black arrowhead) did not label with DAPI, and were distinguishable from hepatic and biliary epithelial nuclei (blue). (B2) Transmission electron micrograph showing cellular changes consistent with hydropic vacuolation, which was observed in both hepatocytes (black arrowhead) and bile preductular epithelia (not shown). Vacuoles ranged from 2 μm to 10 μm in diameter, and were found to be partially to completely filled with electron dense infiltrate. (B3) In vivo confocal image of YO-PRO-1, DIC and TRITC composite: nuclear labeling experiments performed with YO-PRO-1 revealed uptake of YO-PRO-1 into cells with putatively compromised cell membranes. In grayscale DIC image hydropic vacuoles (black arrowhead) are distinct. Associated with hydropic vacuolation was a slight increase in apoptosis (ovate green fluorescence, cell type not known). Of interest is the almost literal appearance of hydropic vacuoles in the confocal DIC fluorescence image (grayscale), where vacuoles appear as liquid droplets. Gill (Gl), Ventral Aorta (Va), Heart Atrium (Ha), Heart Ventricle (Hv), Liver (L), Gall Bladder (GB), Gut (Gt).

**Figure 9:**
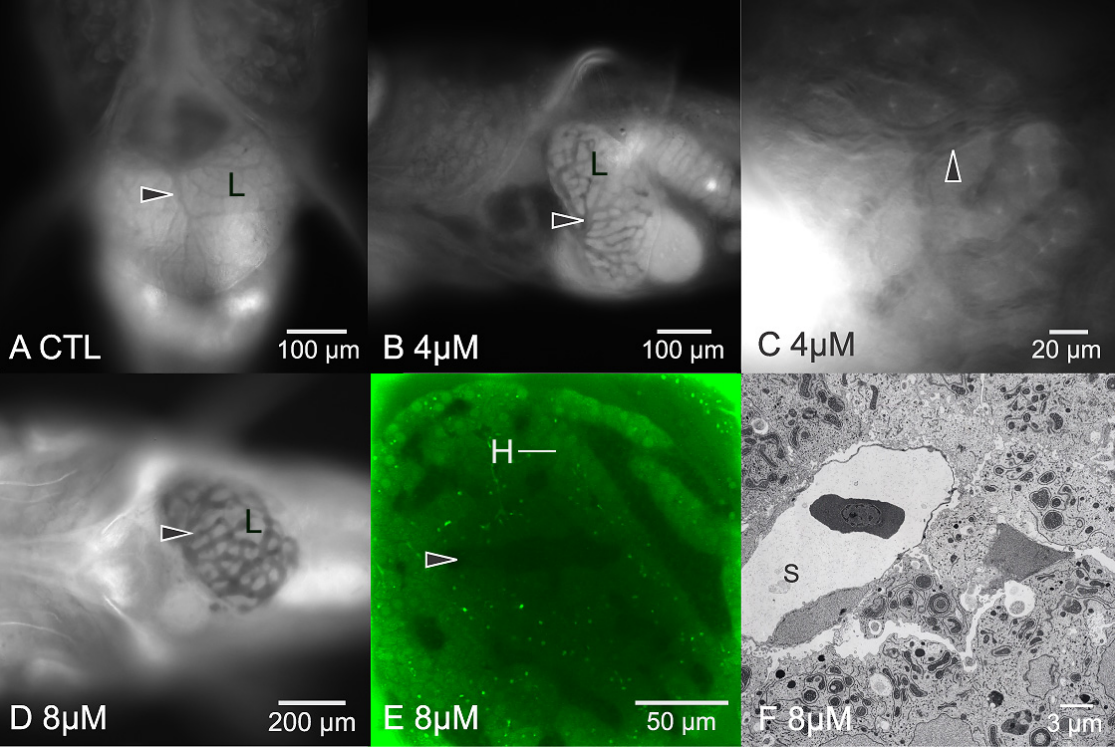
**In vivo imaging of hepatobiliary toxicity: passive hepatic congestion**. (A) Widefield fluorescence DAPI/UV, control liver, 20 dpf, showing the normal in vivo appearance of hepatic vasculature (black arrowhead). Vasculature appears dark (non fluorescent). Epithelium of parenchyma appears light gray. (B and C) At 4 μM ANIT, 24 hrs post exposure, modest dilation of hepatic vasculature was observed throughout the liver (black arrowhead). Image B is DAPI/UV (autofluorescence), image C is TRITC (autofluorescence). (D) At 8 μM ANIT, 24 hrs, marked dilation of the intrahepatic vasculature was observed (DAPI/UV). (E) In vivo confocal imaging, acquired at 48 hrs post ANIT exposure, confirmed dilation of intrahepatic vasculature (black arrowhead) was a pan-hepatic response, occurring uniformly throughout the liver. Vasculature is dark gray, hepatocytes (H) appear green. (F) Transmission electron micrograph (8 μM, 20 dpf, 48 hrs post exposure) of an intrahepatic vessel in ANIT treated medaka, revealing abnormal sinusoid/endothelial cell morphology (S). A single red blood cell can be seen in the sinusoid lumen. Endothelium is highly attenuated. In tandem with morphological changes were changes to cardiovascular function; decreasing heart rate and motility along with increase in vasodilation, in medaka exposed to 0.5 μM to 8 μM ANIT. Control heart rates averaged 129 bpm to 140 bpm (n = 12). Heart rate decreased with increasing ANIT concentrations. At 8 μM ANIT heart rate (means) was observed to be 118 bpm at 6 hrs post exposure, 73 bpm at 24 hrs post exposure, and 61 bpm at 48 hrs post exposure. At dosage regimes above 4 μM (acute and chronic), vasodilation of the hepatic vasculature was evident. Dilation of sinusoids, hepatic vein, and hepatic portal vein, were all observed. Sinusoid diameters: control sinusoids averaged 7.4 μm. At 8 μM ANIT, 48 hrs post exposure, sinusoid diameter averaged 15.3 μm.

Gross anatomical investigation of internal organs and tissues was, expectedly, best imaged with brightfield microscopy (*e.g*., Nikon LSM dissecting microscope), though to some extent widefield microscopy was also successfully applied (see Figures 1, 2, 3, 4 and 5). Both widefield and confocal fluorescence microscopy proved excellent for high resolution (< 1 μm) in vivo observation/imaging of individual cells, tissues and organs in living individuals (Figures 1, 2, 3, 4, 5, 6, 7, 8 and 9). While confocal microscopy was superior to widefield in virtually all aspects, as will be evidenced in the applications described throughout, widefield microscopy was not without merit and should be considered a practical means by which to undertake in vivo investigations in STII medaka. For instance, widefield microscopy was employed as a screening method, and for refinement of cellular level methodologies (*e.g*., screening fluorescent probe efficacy and labeling properties). A fundamental limitation of widefield fluorescence microscopy was a greater depth of field, as compared with confocal. Because of the lack of planar resolution (depth of field permitted with Xenon laser and standard Neo-Fluar objectives, see materials and methods), accurate quantitation of fluorescence was precluded, and widefield fluorescence microscopy was found limited to qualitative studies (vs. quantitative in vivo study/imaging). By example, the depth of field with a 60× Plan Neo-Fluar objective on our Zeiss Axioskopp (widefield) was 0.65 μm; technically, comparable to confocal. However, this 0.65 μm depth of field represents only the field of fine focus. Also resolved, due to illumination by Xenon laser light source, were out of focus planes above and below the plane of focus. Due to the widefield optics and illumination source, reflection and refraction from tissues above and below the plane of imaging resulted in diminished optical resolution, as compared to confocal microscopy. Hence, the optical characteristics of widefield microscopy precluded accurate study of in vivo transport.

LSCM, due to higher planar resolution, was optimal for quantitative investigations into in vivo hepatobiliary transport (see Figures 2, 3, 4, 5, 7 and 8 for comparison). LSCM also offered the advantage of acquisition of confocal stacks of up 100 μm in depth, at planes of section from 0.5 μm to 2 μm. Further, individual confocal stacks could be joined, allowing for final in vivo confocal image stacks of up 300 μm in depth (the maximal depth, we found, at which high quality in vivo imaging could be performed with the system used). In summary, while widefield was sufficient for qualitative study/imaging of hepatobiliary structure, function and transport, accurate quantitative study/imaging required the finer spatial resolution provided by confocal microscopy. Examples illustrating the differences between widefield and confocal microscopy are given throughout the findings presented.

### Fluorescent probes and endogenous fluorophores

The use of fluorescent probes in living medaka greatly enhanced our ability to investigate hepatobiliary structure/function and xenobiotic response in vivo. While fluorescent probes have been widely employed in vitro (*e.g*., cell culture based investigations), few commercially available fluorophores were, at the time these studies were undertaken, characterized for in vivo use in fish. Fluorophores were selected based on their biocompatibility and utility in analysis of hepatobiliary morphology and transport, and are given in Table 1. That a probe would exhibit uptake via aqueous bath was a priority for selection (we preferred to avoid use of intraperitoneal injection).

For brevity, a discussion of fluorescent probes most frequently employed for elucidation of cell/tissue/organ structure and function is given. Metabolic substrates 7-benzyloxyresorufin (7-BR, CYP3A substrate) and 7-ethoxyresorufin (7-ER, CYP1A substrate) were found valuable in vivo probes for investigating CYP3A and CYP1A expression in organs and tissues, and for elucidating the intra- and extrahepatic biliary system, as well as gut lumen. Both probes, non-fluorescent in their native state, are metabolically activated (de-alkylation of 7-BR, de-ethylation of 7-ER) by their respective CYP enzymes to the anionic fluorescent metabolite resorufin. Both probes have been widely employed in vitro [56,57], and 7-ER has been employed in vivo in *Fundulus heteroclitus *and medaka [58-61]. An example of the in vivo application of 7-benzyloxyresorufin, elucidating in vivo CYP3A metabolism, transport, and hepatic morphology, is given in Figure 2.

Sphingolipids are a structurally diverse class of compounds composed of a polar head group and two nonpolar tails (akin to phospholipids), and are naturally occurring compounds found in all plants and animals. As such, we considered the potential for fluorescently-labeled sphingolipids to be incorporated into live cells, and found two fluorescent sphingosines, β-Bodipy C5-HPC and Bodipy FL C5 ceramide, to be useful for in vivo study in STII medaka. β-Bodipy C5-HPC, a fluorescently-labeled phosphocholine, proved biocompatible and was found optimal for elucidating epithelia, endothelia, and hepatobiliary transport. β-Bodipy C5-HPC labeled virtually all epithelia throughout the body of STII medaka. While in vivo kinetics for fluorescently-labeled sphingosines like β-Bodipy C5-HPC and Bodipy FL C5 ceramide are currently unknown, and little information exists on their in vivo metabolic properties, our observations suggest β-Bodipy C5-HPC is recognized as an endogenous sphingolipid, as β-Bodipy C5-HPC was readily incorporated into virtually all cell membranes under aqueous exposure conditions. For instance, β-Bodipy C5-HPC was not only taken up by virtually all epithelia, but also observed to cross the blood brain barrier (the only fluorophore observed to do so), labeling neural bundles in the medulla of medaka (Figure 3). β-Bodipy C5-HPC appeared to be taken up across gill epithelium, and transported through the cardiovascular system to the liver and gut. A small organic cation, the in vivo characteristics of this probe suggested concentrative transport of the fluorophore from blood to bile (hepatic), with fairly rapid (15 minutes post aqueous exposure) accumulation of the fluorophore in bile. While empirical and quantitative studies suggest concentrative vectorial transport of this fluorophore, in vivo kinetics will need further study and confirmation.

The in vivo kinetics of Bodipy FL C5 ceramide appeared more consistent with passive diffusion across cell membranes. Uptake and distribution of the fluorophore was observed to be substantially slower (30 – 45 minutes slower) than β-Bodipy C5-HPC. Where the fluorescent phosphocholine saw uptake and concentration in intrahepatic biliary passageways as early as 15 minutes post administration (aqueous bath), the fluorescent ceramide was not observed to accumulate (peak fluorescence) in the hepatobiliary system until ~45 minutes post exposure.

Cardiovascular transport of Bodipy FL C5 ceramide was much more distinct than that of β-Bodipy C5-HPC, with marked and prolonged residence time in blood plasma, and distinct labeling of endothelium and red blood cells. Bodipy FL C5 ceramide, because of its apparently slower (passive?) uptake, and accumulation in the cytosol of virtually all cell types, was found optimal for investigating epithelial cell morphology in vivo. This fluorophore also exhibited a more even distribution (concentration) across blood plasma, cytosol, and intrahepatic bile passageways. Hence, Bodipy FL C5 ceramide proved highly effective for elucidating cell morphology in vivo, allowing imaging of sinusoids, hepatocytes, and intrahepatic biliary passageways, simultaneously.

The highly lipophilic Bodipy 505/515 and 493, like β-Bodipy C5-HPC, were also found to be effective fluorophores for investigating hepatobiliary transport and bile secretion in vivo (Figure 3). Both probes allowed elucidation of the intrahepatic biliary passageways, extrahepatic biliary passageways, gut lumen, and intestinal lumen (*e.g*., mucosal folds of the gut).

Fluorescein isothiocyanate (FITC) was also utilized for elucidating the biliary architecture. This small organic cation showed rapid uptake, with marked differential distribution between blood and bile, and transport into biliary passageways. Because of the difference in concentration of FITC (as well as other fluorophores) in blood and bile on a temporal scale, this allowed for high resolution in vivo investigation into biliary morphology, bile transport, and xenobiotic response. (Note: the temporal variation in blood and bile concentrations is the result of fluorophore uptake and transport kinetics, determined by the physical and chemical properties of the fluorophore, and passive and active transmembrane transport kinetics in gill epithelia and hepatocytes.)

Several probes were found to be useful for in vivo nuclear labeling, these were: DAPI 4',6-diamidino-2-phenylindole, dihydrochloride; DAPI Diacetate 4',6-diamidino-2-phenylindole, diacetate; and the SYTO^® ^and YO-PRO^® ^series of fluorescent probes from Invitrogen (Figure 8). The ability to label and visualize nuclei of individual cells in organs and tissues proved valuable not only for structural elucidation (particularly in 3D), but for differentiation between nuclei and cellular organelles (described later in the xenobiotic response section).

In addition to the utilization of exogenous fluorophores, utilization of auto-fluorescence (an innate property of live cells) of cells was highly useful in delineation of cellular and tissue morphology in vivo (Figures 2, 4, 5, 8, 9). A variety of endogenous compounds are known to fluorescence, and the excitation/emission spectra of some of these are given in Table 2. Among them, endogenous fluorescence of nicotinamide adenine dinucleotide phosphate (NADPH), riboflavin, flavin co-enzymes and flavoproteins, as well as porphyrins, has been used as an indicator of enzyme activity and physiological state in living cells [62]. For instance, NADPH fluoresces in its reduced state, but not in its oxidized state (*e.g*., NADP). In contrast flavin adenine dinucleotide fluoresces in its oxidized state (FAD), while fluorescence is undetectable when reduced to FADH_2_. Because each of these endogenous fluorophores are widely employed in a variety of cellular processes, they, by their fluorescence, can be good indicators of cell viability and function. For instance, Ramanujam et al (1994) reported that dysplastic cells exhibited greater fluorescence, likely due to increased metabolic rate of altered cell types [63]. These studies revealed UV light excitation was effective for differentiation of neoplastic and normal cervical tissue, in vivo [63]. Similarly, differential fluorescence has been employed to distinguish normal, hyperplastic, and adenomatous human colonic epithelia cells of the mucosa (primary cell cultures) [64]. Our own in vivo observations noted that the autofluorescence of non viable cells and tissues was diminished relative to their viable counterparts.

**Table 2:** Endogenous fluorophores.

	**Ex (nm)**	**Em (nm)**
Flavoproteins: flavin adenine dinucleotide (FAD), flavin mononucleotide (FMN)	450	550
Pyridine Nucleotides: nicotinamide dinucleotide (NAD), nicotinamide dinucleotide phosphate (NADP), the reduced states fluoresce – NADPH, NADH	336	450
Tryptophan	295	329
Hydroxykynurenine glucoside (3-HGK)	520	550
Serotonin (5-HT)	290/366	340-440/510
5 HIAA	300	350
α-napthol	340	460
Bilirubin	435	500
Protoporphyrin	424/408	594/630
Collagen I	340/500	410/520
Elastin	330	405
Lipofuscin	300	420
Vitamin A	345	490
Vitamin E	295	335
Thiamin (B1)	366	430
Riboflavin (B2)	440	514
Salicylic Acid	319	408

Given are some of the known endogenous fluorophores and their excitation (Ex) and emission (Em) wavelength(s).

Our studies found UV/DAPI excitation to be highly useful in elucidation of morphological features, otherwise difficult to visualize. For instance, UV/DAPI excitation was highly effective for visualizing vasculature (blood plasma was relatively non fluorescent compared to individual epithelial, endothelial and red blood cells). UV/DAPI excitation was also useful for visualizing individual epithelial cells in vivo (*e.g*., hepatocyte morphology). Hence, the innate autofluorescence properties of cells/tissues can be utilized for investigation of a variety of structural and functional, as well as diagnostic purposes. Further, savings in time and materials may be realized, negating the need to administer exogenous fluorophores to elucidate cellular and tissue morphology. Note however that UV excitation wavelengths (< 400 nm) are known to be cytotoxic and special care must be taken when employing these methods.

At other excitation/emission wavelengths (TRITC/FITC) general tissue autofluorescence was less distinct than that observed with DAPI/UV. However, autofluorescence of bile fluid was marked under both TRITC and FITC illumination. Because of this TRITC/FITC excitation/emission could be employed to detect the onset of bile synthesis (endogenous bile fluorescence), and to observe bile transport in vivo. Of interest, we observed bile autofluorescence to vary on a temporal scale, a fact which merits further detailed study. While TRITC/FITC excitation resulted in distinct autofluorescence of intra- and extrahepatic bile passageways, as well as gall bladder, it was not consistently observed, suggesting variations in bile composition, perhaps due to varying concentrations of endogenous fluorophores (in bile) previously discussed (*e.g*., NADP, FADH, porphyrin compounds). For this reason, it was not possible to fully utilize bile autofluorescence as an indicator of hepatobiliary transport of specific bile constituents, though significant potential exists in applying fluorescence studies to the examination of bile transport.

### Quantitation of hepatobiliary transport in vivo

For accurate quantitation of transport several key elements were necessary, and merit a brief discussion. One of the primary issues was standardization of microscopy techniques [65-67]. Exposure times while imaging (*e.g*., fluorophore quenching), laser intensity (excitation), fluorophore concentration in the exposure bath, and imaging depth in vivo (*e.g*., how deep into the tissues you are imaging), were each found to influence and define the digital image information captured in vivo, and this, is turn, affected quantitation of digital information. Perhaps one of the most important factors was the time of image acquisition. Most fluorophores used for investigation of blood to bile transport were observed to be assimilated by medaka fairly rapidly (10–20 minutes for initial uptake from aqueous bath). Peak/maximal fluorescence (a proxy for fluorophore saturation) occurred some 15 to 90 minutes later, depending on the fluorophore used. Hence, in developing in vivo methodology for quantifying hepatobiliary transport, several key elements were/are required. First, uptake, distribution and transport of individual fluorophores should be fully characterized for standardization of protocols. This would entail characterizing temporal changes, as well as the effect of fluorophore concentration (aqueous bath), on uptake and transport kinetics. In our investigations rigorous study and thorough determination of fluorophore kinetics in vivo was not possible due to the lack of a dedicated confocal instrument, and time. In lieu of this type of characterization, observed peak fluorescence times, and a time frame subsequent to peak fluorescence that allowed for in vivo imaging and quantitation of transport, was determined for each fluorophore employed.

We provide in Table 1 times at which fluorescence was first observed in the biliary passageways of the liver (initial fluorophore uptake), and the time when fluorescence peaked (maximal saturation). After peak fluorescence was achieved, fluorophore concentration in vivo (fluorescence) was assumed to be at a steady state as long as medaka remained under constant exposure to the fluorophore in aqueous medium. At this point, transport kinetics were assumed to have reached equilibrium (at the exposure concentrations given, under chronic exposure conditions). For our studies it was decided image acquisition would occur from the onset of peak fluorescence (saturation), or the time when fluorescence intensity peaks at a given exposure concentration (unique for each fluorophore), and for a predefined period of time following the onset of peak fluorescence; for our studies, 60 minutes. Hence, imaging was done at the onset peak fluorescence and for, on average, no more than 60 minutes post peak fluorescence. With the above factors accounted for, we found in vivo quantitation of hepatobiliary transport possible and a potentially valuable diagnostic tool.

For quantitation, digital images from in vivo investigations were converted to both RGB 32 bit color, and 8 bit grayscale. These were then analyzed in ImageJ using the following techniques. Regions of interest (ROIs) were defined for sinusoid lumen, hepatocyte cytosol, and canalicular/bile preductular lumens. ROIs were measured for fluorescence intensity (brightness value for RGB and grayscale value for grayscale) in both RGB 32 bit color, and 8 bit grayscale images. In the image under study multiple ROIs were randomly selected for; sinusoidal space, cytosol space and bile space. For instance, in a confocal stack, acquired from in vivo imaging, multiple ROIs were measured in each of the three compartments, resulting in repeated measures. Fluorescence intensity values, as well as the total area measured for sinusoid, cytosol and bile space, were imported to Excel and Statview for statistical analyses. Means were taken for each set of measures (sinusoid, cytosol, bile space) and used for plots/graphs. Descriptive statistics as well as bivariate analyses were used for quantitation.

### Three-dimensional reconstructions from in vivo imaging

The fluorescent probes 7-benzyloxyresorufin (7-BR), β-Bodipy C5-HPC, Bodipy FL C5-ceramide, DAPI, and fluorescein isothiocyanate (FITC) were administered to living STII medaka in aqueous bath (see Table 1) to elucidate specific components of the hepatobiliary system (*e.g*., hepatocytes, endothelial cells, biliary epithelia, bile passageways). Individual medaka were exposed to fluorophores (aqueous bath) at the following concentrations and durations: 7-BR (10–50 μM, 10 to 30 minutes), β-Bodipy C5-HPC (30 nM–10 μM, 10 to 30 minutes), Bodipy FL C5-ceramide (500 nM–5 μM, 10 to 44 minutes), FITC (1 nM – 50 μM, 10 to 30 minutes). After 15 – 60 minutes of fluorophore exposure STII medaka were sedated, mounted on depression well glass slides with cover slip and imaged live using LSCM, at various stages of development (4–60 dpf). Confocal stacks from in vivo imaging of the hepatobiliary system were then imported into the 3D rendering and analytical software, Amira 3D. Confocal stacks were comprised of 0.5 μm to 2 μm slices (space between individual images/scans), though 0.7 μm was most commonly employed. Stacks were typically 90 to 120 μm thick (depth of scan), though in some instances these were combined to create stacks of up to 200 μm in thickness. The 3D reconstructions, manually created in Amira 3D, were then used for architectural, morphometric and volumetric analyses.

## Results and discussion

### In vivo description of hepatobiliary development

In vivo investigations permitted detailed study and description of normal development of the medaka hepatobiliary system, which included observation/description of hallmark events such as organogenesis, the onset of bile synthesis and transport, and metamorphosis of the liver from an embryonic to adult phenotype (Figure 4). In vivo investigations also permitted direct observation of hepatic architecture at the cellular level at various developmental stages, observations which, coupled with 3D reconstructions of the liver, led to important insights. For instance, characterization of variations in parenchymal architecture between embryonic and larval stages, and the comparative similarities and differences in vertebrate liver conceptual models [12]. Figures 2, 4 [and Additional files 1, 2, 3, 4, 5, 6, 7, 8 and 9] provide examples of results from in vivo investigations into hepatobiliary development (unpublished studies), investigations that were fundamental to characterizing normalcy (in vivo) and to subsequent investigations into adaptive *vs*. toxic responses of the liver to xenobiotic exposure (see *In vivo investigation of xenobiotic response*).

### Quantitation of hepatobiliary transport in vivo

To put these studies into context a brief overview of hepatobiliary transport is provided. Transport of solutes from blood to bile is a vital liver function. It is through bile synthesis and transport that xenobiotics of environmental origin and endogenous metabolic by-products are either safely removed from the system, or, with systemic accumulation, result in morbidity and mortality [4,68-74]. Inhibition/impairment of bile transport (cholestasis) commonly results in morbidity and mortality in mammals. Little is known about bile transport in piscine species, and the relationship of impaired transport function to disease and toxicity in these organisms. While it is becoming increasingly apparent that many piscine species share bile synthetic and transport mechanisms with their mammalian counterparts [14,75,76], no studies on impaired/inhibited bile transport (cholestasis) in fish exist. Here we show methods that can be used to perform in vivo assessment of bile transport, and present initial findings employing these methodologies.

Our investigations show in vivo quantitation of hepatobiliary transport in STII medaka is possible, and a potentially valuable diagnostic tool for evaluation of normalcy and toxic response (*e.g*., cholestasis) in this animal model. Using fluorescent probes success was achieved in evaluating blood to bile transport in vivo under both conditions of normalcy and toxicity in STII medaka. Examples are given in Figures 5 and 6 and Table 3. While differences between Bodipy C5 ceramide, and β-Bodipy C5-HPC and fluorescein isothiocyanate transport were expected, the more subtle, statistically significant differences between β-Bodipy C5-HPC and fluorescein isothiocyanate (Figure 6, Table 3) were especially interesting. For instance, in contrast to C5 ceramide, which exhibits kinetics more consistent with passive diffusion across cell membranes, the latter two fluorophores exhibited kinetics more consistent with active transport. That these differences are putatively indicated here in quantitative results is promising for future investigations into quantifying transport of solutes from blood to bile in vivo.

**Table 3:** Quantitative analysis of blood to bile transport in vivo (STII medaka, 12 dpf): β-bodipy C5 phosphocholine (HPC), fluorescein Isothiocyanate (FTIC) and bodipy C5 ceramide.

	F-test: Hypothesized Ratio = 1					
	Canaliculus		Cytosol		Sinusoid	

	F-value	P-value	F-value	P-value	F-value	P-value
C5 Ceramide: FITC	1.026	0.9665	38.508	< .0001	18.672	0.0001
C5 Ceramide: HPC	0.117	0.0019	17.109	0.0002	28.413	< .0001
FITC:HPC	0.114	< .0001	0.444	0.0686	1.522	0.3412

Equality of variance F-test comparing measured values (repeated measures, which varied from 30 to 70 depending on number of stacks and available fields) among cytosol, sinusoid and canalicular spaces are given with P-values. See also Figure 6.

### Three-dimensional in vivo investigations: hepatobiliary architecture

Non invasive in vivo imaging in STII medaka allowed the generation of 3D models of the hepatobiliary system (Movies 1 – 9), under conditions of normalcy and toxicity. Using LSCM, in tandem with exogenous fluorophores, we were able to elucidate hepatocellular, biliary and vascular components of the liver in 3D. Three-dimensional investigations yielded important insights into medaka hepatobiliary structure/function, which may not have been possible using standard 2D methodologies alone (*e.g*., histological, ultrastructural). For instance, 3D analyses revealed that: the hepatic parenchyma in medaka is organized through a hexagonal structural motif (polyhedral tessellation), evident in the fine structure of the biliary system; the biliary system is an interconnected network of canaliculi and bile preductules; the canaliculo-preductular network perfuses the majority of the liver corpus (~95%) uniformly, with equidiameter intrahepatic biliary passageways (IHBPs) (1–2 μm) observed throughout the liver; larger bile ductules and ducts were observed only at the liver hilus, and consequently an arborizing biliary tree was absent, seen only in the rudimentary branching of intrahepatic ducts from the hepatic duct; parenchymal architecture is a predominantly 2 cell thick muralium, though tubule-like formations may also comprise the muralium; the livers of these small fish are replete with BPDECs, the putative mammalian correlary of bipotential progenitor/stem cells; BPDECs and hepatocytes form unique junctional complexes that create bile passageways (bile pre-ductules). BPDECs occupy the center of these junctional complexes, surrounded by bile pre-ductules. Collectively, these findings characterized the 3D architecture of the medaka hepatobiliary system and improve our comparative understanding of vertebrate liver structure and function. These findings also raised interesting questions regarding the "functional unit" of the vertebrate liver, suggesting the hepatobiliary system in medaka can be, as a conceptual model, considered a single functional unit of the vertebrate liver, akin to an individual unit of the mammalian lobule [12].

Analyses of 3D reconstructions also provided highly accurate volumetric and ratiometric information on biliary, parenchymal, vasculature and liver volumes, under both conditions of normalcy and toxicity (Tables 4, 5, 6 and 7). In vivo morphometric and volumetric findings were found to correlate well with prior ex vivo studies in vertebrate livers (Table 6), though in vivo findings see marginally higher hepatocellular and lower vascular volumes. Hence, it is possible that ex vivo findings may underestimate the volumetric indices of these compartments to some degree, given in vivo studies, which capture fully perfused organs (*e.g*., cytosol, vasculature), likely generate more realistic indices. While our focus was the hepatobiliary system, it should be evident from the examples presented here, and previously [12], that these types of 3D investigations in STII medaka, using the same or similar methodologies, are possible in other organ systems as well.

**Table 4:** Hepatobiliary morphometrics from in vivo based 3D investigations.

**Biliary**		**Mean**	**Mode**	**SD**	**Min**	**Max**		**n **=
**8 dpf**	**IHBP Segment Length **(μm)	11.8	11.6	1.2	10.0	17.0		68.0
**12 dpf**		11.9	11.6	1.4	9.2	15.8		39.0
**30 dpf**		12.8	11.3	1.6	9.8	16.5		34.0
**40 dpf**		11.6	11.1	1.3	8.5	14.6		42.0
								
**8 dpf**	**Degree 120**	119.8	118.0	6.7	100.0	137.0		81.0
**12 dpf**		119.8	127.0	7.2	103.0	136.0		61.0
**30 dpf**		117.2	123.0	20.8	11.5	134.0		33.0
**40 dpf**		124.4	123.0	8.5	103.0	146.0		73.0
								
**8 dpf**	**Degree 90**	91.4	91.0	4.2	83.0	99.1		15.0
**12 dpf**		89.9	89.5	5.1	80.6	98.8		16.0
**30 dpf**		91.0	-	8.0	78.0	101.0		6.0
**40 dpf**		98.5	103.0	7.9	84.4	109.0		12.0
								
**8 dpf**	**Degrees Other**							
**12 dpf**		56.6	50.0	14.7	44.0	88.0		15.0
**30 dpf**		68.7	40.0	37.6	40.0	163.0		10.0
**40 dpf**		66.4	49.0	32.6	41.0	154.0		9.0
								
**8 dpf**	**IHBP Diameter **(μm)	1.3	1.3	0.3	0.9	1.8		30.0
**12 dpf**		1.4	1.4	0.2	1.0	2.0		37.0
**30 dpf**		1.3	1.2	0.3	0.9	2.2		22.0
**40 dpf**		1.3	1.3	0.5	0.5	2.6		23.0
								
**8 dpf**	**BPD Segment Length **(μm)							
**12 dpf**		6.2	6.3	1.5	3.8	9.4		15.0
**30 dpf**		7.4	6.2	2.6	3.9	13.5		15.0
**40 dpf**		8.3	-	2.5	4.3	15.6		21.0

**Cellular**								

**8 – 40 dpf**	**Hepatocyte Diameter **(μm)	11.3	11.8	1.0	8.4	13.5		48.0
								
**8 – 40 dpf**	**BPDEC **(μm)	5.5	4.3	1.3	3.3	10.2		48.0
								
**8 dpf**	**Muralium/Tubule Diameter ** (height variance of 20 μm to > 100 μm)	21.2	22.7	3.6	13.5	32.7		36.0
**20 dpf**		29.6	34.5	8.6	19.8	63.4		38.0
**30 dpf**		26.0	26.9	5.7	17.3	43.8		32.0
**40 dpf**		24.8	24.5	6.3	16.9	48.7		36.0

**Vasculature**								

**8 dpf**	**Sinusoid Diameter **(height variance of 8 μm to 31 μm)	6.9	6.7	0.9	5.2	8.8		26.0
**20 dpf**		7.5	6.9	1.3	5.4	11.3		32.0
**30 dpf**		7.6	7.6	1.6	4.9	12.1		32.0
**40 dpf**		7.9	7.1	1.9	3.3	12.6		34.0
								
**20 dpf**	**Hepatic Vein Diameter **(μm)	18.9						
**30 dpf**		18.3						
								
**20 dpf**	**Portal Vein Diameter **(μm)	13.9						
**30 dpf**		14.7						

Metrics obtained from 3D reconstructions of the hepatobiliary system at 8, 12, 20, 30 and 40 dpf provided highly accurate morphological assessment of components of the hepatobiliary system. IHBP segment length corresponds to average length of the canaliculus, which was found to be approximately the same as hepatocyte diameter. Bile preductule (BPD) segment length is length of bile passageway surrounding BPDECs, a length that approximates BPDEC diameter. In vivo indices from 3D analyses correlated will with ex vivo ultrastructural (TEM) indices, and in vivo 2D metrics.

**Table 5:** Hepatobiliary volumetrics from in vivo based 3D investigations.

**8 dpf**	**Volume μm**^3^	**% Volume**	**SA μm^2^**
Intrahepatic Biliary Passageways	3653	1.03%	12259
Vasculature	22416	6.33%	23586
Parenchyma	331569	93.67%	
Hepatocellular	327916	92.64%	
Liver Corpus	353985	-	47310
			
**Stack size: **115 × 115 × 50 μm, sections: 1 μm, scaling/voxel size: 0.2 × 0.2 × 1 μm, 3D volume analysis: ~50% total liver volume			

**12 dpf**	**Volume μm**^3^	**% Volume**	**SA μm**^2^

Intrahepatic Biliary Passageways	19877	0.86%	47808
Vasculature	199157	8.60%	127215
Parenchyma	2117236	91.40%	
Hepatocellular	2097359	90.54%	
Liver Corpus	2316393	-	318879
			
**Stack size**: 230 × 230 × 90 μm, sections: 1 μm, scaling/voxel size: 0.45 × 0.45 × 1 μm, 3D volume analysis: ~50% total liver volume			

**30 dpf**	**Volume μm**^3^	**% Volume**	**SA μm**^2^

Intrahepatic Biliary Passageways	11435	1.01%	24871
Vasculature	85837	7.60%	38682
Parenchyma	1043352	92.40%	
Hepatocellular	1031917	91.39%	
Liver Corpus	1129189	-	272427
			
**Stack size**: 325 × 325 × 178 μm, sections: 1 μm, scaling/voxel size: 0.64 × 0.64 × 2 μm, 3D volume analysis: ~15% total liver volume			

Hepatobiliary metrics obtained from 3D reconstructions of the hepatobiliary system at 8, 12 and 30 dpf. A consistent relationship among the volume of intrahepatic biliary passageways, vasculature, hepatocellular volume, and parenchymal volume, in relation to total liver volume analyzed (liver corpus), was found across each stage of development. Given are the dimensions of in vivo confocal stacks from which 3D reconstructions were made, as well as voxel size (a proxy for resolution). Also given are estimates for the total volume of liver analyzed; for instance, at 8 dpf, it is estimated that the stack size was ~50% of total liver volume, and at 30 dpf, the stack dimensions are estimated to comprise ~15% of total liver volume. Total volume of each 3D reconstruction corresponds to volume of liver corpus. Parenchymal volume is comprised of hepatocellular space, BPDECs and intrahepatic biliary passageways. SA = Surface Area; Vol = Volume

**Table 6:** Comparative hepatobiliary volumetric studies.

**Component (% Volume)**	**Rat **[78]	**Dog **[79]	**Rainbow trout **[80]	**Golden Ide **[81]	**Brown trout **[16]	**Medaka**
Hepatocytes	77.8	84.4	84.5	88.9	87.3	91.5
Biliary Epithelia	22.2	15.6	15.5	11.1	15.18	-
Sinusoid Lumen	10.6	4.3	9.4	6.6	6.23	7.51
Bile Canaliculi	0.4	0.5	1.1	0.7	0.45	0.96

Given are comparative metrics for components of the hepatobiliary system from mammalian and piscine studies. When comparing metrics note that values for medaka are from 3D in vivo investigations, metrics from other studies were derived from ex vivo histological and ultrastructural studies. No total volume was obtained for biliary epithelia in medaka, however, these cell types were found predominantly in the hilar region of the liver, and it is estimated they comprise a similar volume as those reported in other studies above.

**Table 7:** Volumetric assessment of α-napthylisothiocyanate (ANIT) exposed livers

**30 dpf 2.5 μM ANIT, 48 hpd**	**Volume μm**^3^	**% Volume**	**SA μm**^2^
Intrahepatic Biliary Passageways	11743	0.5%	37818
Vasculature	159187	7.3%	92092
Parenchyma	2023674	92.7%	0
Hepatocellular	2011931	92.2%	0
Liver Corpus	2182861	-	156422
Stack size: 230 × 230 × 89 μm, 1 μm sections, scaling/voxel size 0.45 × 045 × 1 μm, 3D volume analysis ~30% total liver volume			

**30 dpf 1 μm ANIT, 48 hpd**	**Volume μm**^3^	**% Volume**	**SA μm**^2^

Intrahepatic Biliary Passageways	100354	1.18%	164119
Vasculature	421852	7.92%	207939
Parenchyma	8083460	92.08%	
Hepatocellular	7983106.00	100.00%	
Liver Corpus	8505312		546491
Stack size: 325 × 325 × 89 μm, 1 μm section, scaling/voxel size 0.64 × 0.64 × 1 μm. ~30% total liver volume			

	Mean: 8 – 30 dpf	2.5 μM ANIT 30 dpf	1 μM ANIT 30 dpf
**CTRL vs. Treated**	**% Volume**	**% Volume**	**% Volume**

Intrahepatic Biliary Passageways	0.97%	0.5%	1.18%
Vasculature	7.51%	7.3%	7.92%
Parenchyma	92.49%	92.7%	92.08%
Hepatocellular	91.52%	92.2%	92.15%
Liver Corpus			

Volumetric comparisons between normal and ANIT exposed livers. Indices are summarized in the lower part of Table 7 (see also Table 5). Medaka treated with 1 μM ANIT and analyzed (3D reconstructions, volumetrics) at 30 days exhibited an increase in volume of intrahepatic biliary passageways. Medaka treated with 2.5 μM ANIT and analyzed at 30 days exhibited a decrease in intrahepatic biliary volume. Where biliary volumes were changed in ANIT exposed animals, other indices of vasculature, parenchymal and hepatocellular volume remained relatively well conserved across normal and treated medaka. These results, while from only 2 treated animals, may suggest a choleretic response at low ANIT exposure concentrations, and cholestatic like response at higher aqueous ANIT concentrations. Due to the time consuming nature of 3D reconstructions, only 2 livers of treated animals were reconstructed in full.

### In vivo investigation of xenobiotic response

With normalcy characterized (in vivo phenotypes of cells/tissues, hepatobiliary development, 3D parenchymal architecture, evaluations of blood to bile transport), it was then possible to apply this experimental system to the investigation of toxic response in vivo (see also [77]). To do so we used two reference hepatotoxicants; α-napthyl isothiocyanate (ANIT) and diethylnitrosamine (DEN). In vivo investigations (responses to xenobiotic exposure) were correlated with histopathology, ultrastructure and immunohistochemistry for validation/characterization of xenobiotic response/toxicity.

An example of non invasive in vivo serial analysis of the adult consequence of early life stage exposure to DEN is given in Figure 1. Shown is an in vivo assessment of neoplastic response 10 months post exposure to DEN. Histopathology revealed the tumor to be comprised of mixed neoplasms of hepatocellular and biliary origin, and with foci of biliary hyperplasia.

In vivo evaluation of responses of the liver to ANIT exposure revealed distinct dose dependent phenotypic changes, these included: (1) canalicular attenuation and dilation in response to 1 – 3 μM acute aqueous ANIT exposure; (2) bile preductular lesions in response to 2 – 5 μM chronic ANIT exposure; (3) hydropic vacuolation, at ANIT concentrations of 2 – 8 μM ANIT, which resulted in a distinct "pebbling" of the liver when evaluated in vivo; and (4) chronic passive hepatic congestion, an end stage response of the liver associated with high mortality, at 6 – 8 μM ANIT (see Figures 7, 8 and 9). In vivo observations were correlated with ex vivo histological and electron microscopic studies to aid in interpretation of in vivo findings and to verify affected cell types. Lastly, volumetric analyses of 3D reconstructions from ANIT treated medaka suggested a possible reduction in bile flow, as well as choleresis, with no changes to other volumetric liver indices (Tables 4, 5 and 7). In vivo 3D morphometric and volumetric indices were consistent with both in vivo (2D) and ex vivo findings (ultrastructural studies), revealing accuracy and standardization of quantitative assessments across in vivo and ex vivo techniques. These findings, while largely descriptive, suggest ANIT induced changes in the medaka hepatobiliary system are: (1) similar to ANIT induced changes described in rat liver, and (2) consistent with responses commonly observed in cholestasis in mammalian livers.

## Conclusion

We have described the development and application of non invasive in vivo methodologies to the study of biological structure, function and xenobiotic response in STII medaka. The development of this in vivo investigatory "system" encompassed the validation and application of exogenous fluorescent probes, and endogenous fluorescence, for the in vivo study of biological structure, function, and xenobiotic response, in STII medaka, in both 2D and 3D contexts. With confocal microscopy, high resolution (< 1 μm) in vivo imaging was achieved (*e.g*., subcellular).

Of particular interest is the ability to investigate biological structure/function relationships, and xenobiotic response, in 3D, and to potentially integrate molecular mechanisms of toxicity with system level phenotypic changes, in a 3D context. Significant advances have been achieved over the last decade in 3D elucidation of structure/function relationships at the molecular/protein levels of biological organization. However, similar information on organ system structure and function at the cellular level has lagged. Elucidation of biological structure/function relationships in a real world 3D context is vital to advance our interpretive and diagnostic capabilities (*e.g*., normalcy and disease/toxicity), further our comparative understanding of organ system ontogeny, and integrate genetic and molecular information with system levels of biological organization. The latter is of particular importance. One of the main challenges facing life sciences is the integration and interpretation of genomic, proteomic, and metabolomic information in relation to the complex physiological system in which "omic" mechanisms operate. As these findings suggest, STII medaka provide a unique means by which to potentially integrate mechanisms of toxicity (*e.g*., genomic, proteomic function) with system level responses and phenotypic changes, in vivo (the overarching goal of our laboratory).

The findings presented also demonstrate in vivo quantitation of hepatobiliary transport is possible, suggesting STII medaka provide a novel means by which to investigate piscine hepatobiliary transport, and the effects of toxins/toxicants, as well as genetic disorders, on biliary transport mechanisms. Because hepatobiliary transport may be impaired by a variety of pharmaceuticals and environmental contaminants, the ability to investigate altered organ system function in vivo is a valuable tool that should prove valuable to the future study of piscine biliary transport.

Collectively these findings demonstrate the ability to study, with high resolution, normalcy and disease/toxicity in vivo in the hepatobiliary system of living medaka, a capability that provides a valuable diagnostic and investigatory tool. The reviewed findings presented here, in conjunction with earlier studies [11,16,77-85], have, we feel, significantly advanced our comparative understanding of the piscine liver, and we consider the potential for discovery, within the context of in vivo investigation in STII medaka, as significant.

## Competing interests

The authors declare that they have no competing interests.

## Authors' contributions

RCH carried out the majority of research: in vivo methodology development, application of in vivo methodologies to investigation of normalcy and toxicity in STII medaka, and 3 dimensional reconstructions and analyses. SWK provided much appreciated assistance with fluorescent cytochrome P450 substrates. DEH provided invaluable expertise on interpretation of toxicity, histology and ultrastructural work. All authors have read and approved the content of the manuscript.

## Supplementary Material

Additional file 1 **STII medaka, 12 dpf, left lateral view**. Lacking dermal and visceral pigmentation, internal organs are readily visible through the body wall, and amenable to in vivo observation/imaging. Note peristalsis in the gut on a temporal scale. Liver (L), Gut (Gt), Otic Vesicle (Ov), Spleen (Sp), Air/Swim Bladder (AB), Heart (H). Click here for file

Additional file 2 **In vivo confocal imaging of hepatobiliary system, STII medaka, 9 dpf**. Example of a confocal stack acquired in vivo. Parenchyma elucidated here with β-Bodipy C5 ceramide. Hepatocyte nuclei (HN) appear dark (non fluorescent), cytosol is distinct. Red blood cells can be seen in circulation through sinusoids (S/r). Note differential fluorescence between sinusoid lumen vs. cytosol. Endothelial cells lining sinusoids are also distinct. Stack size [x : y : z = 192 × 192 × 62 μm], Scaling [0.37 × 0.37 × 0.7 μm]. Click here for file

Additional file 3 **Three-dimensional reconstruction of canaliculi and sinusoids from in vivo confocal image stack: relationship of sinusoids to intrahepatic biliary passageways, STII medaka, 30 dpf**. Shown is an isolated section of the parenchyma from a 3D reconstruction. Canaliculi (C, green), which average 1.3 μm in diameter, are green, sinusoids (S, red). Examples of metrics acquired from 3D reconstructions are given for illustrative purposes. The example movie shown here, extracted from an Amira 3D reconstruction, is limited to rotation in a single plane. Actual 3D reconstructions can be rotated in any plane, at virtually any magnification, allowing detailed study of hepatobiliary structure/function relationships. Click here for file

Additional file 4 **Example of 3D reconstruction of hepatic parenchyma from in vivo confocal image stack: bile preductules and preductular epithelial cells, STII medaka, 24 dpf**. Shown is an isolated section of the parenchyma showing the 3D characteristics of bile preductular epithelia (BPDEC) and bile preductules (BPD), the latter a unique morphological feature created by junctional complexes between hepatocytes and BPDECs. Hepatocytes, which occupy the negative/empty space, are not rendered for visual clarity. A canaliculus (C, green) is shown joining a bile preductule (BPD, green). The background grayscale image is a single optical section from a confocal image stack. Red blood cells can be seen in circulation through sinusoids of the liver (S/r) in confocal image. To our knowledge this was the first rendering of this bile preductule junctional complex in 3D, the evaluation of which provided novel insights into parenchymal organization. The movie given here, extracted from an Amira 3D reconstruction, is limited to rotation in a single plane. Actual 3D reconstructions can be rotated in any plane, at virtually any magnification, allowing detailed study of hepatobiliary structure/function relationships. Click here for file

Additional file 5 **Example of 3D reconstruction of parenchyma from in vivo confocal image stacks: relationship of sinusoids to intrahepatic biliary passageways, STII medaka, 30 dpf**. Sinusoids (S) are red, canaliculi (C) in green. All space between sinusoids and surrounding canaliculi (empty) is hepatocellular space, not rendered for visual clarity. Morphometric and volumetric analyses of 3D reconstructions assisted elucidation of parenchymal architecture, and relationship of canaliculi to sinusoids. These types of investigations revealed medaka hepatic parenchyma to be more akin to a muralium like structure (as opposed to tubular architecture). Grayscale confocal image can be seen in the background. The movie given here, extracted from an Amira 3D reconstruction, is limited to rotation in a single plane. Actual 3D reconstructions can be rotated in any plane, at virtually any magnification, allowing detailed study of hepatobiliary structure/function relationships. Click here for file

Additional file 6 **Example of 3D reconstruction of hepatobiliary architecture from in vivo confocal image stack: sinusoids and parenchymal architecture, STII medaka, 30 dpf**. Sinusoids (S) are red. All space between sinusoids (empty) is hepatocellular space, not rendered for visual clarity. Morphometric and volumetric analyses of 3D reconstructions allowed elucidation of parenchymal architecture, and revealed medaka parenchyma more akin to a muralium like structure (as opposed to tubular architecture). Background grayscale image is a single frame from a confocal image stack from which the 3D model was generated. The movie given here, extracted from an Amira 3D reconstruction, is limited to rotation in a single plane. Actual 3D reconstructions can be rotated in any plane, at virtually any magnification, allowing detailed study of hepatobiliary structure/function relationships. STII medaka, 30 dpf. Click here for file

Additional file 7 **Example of 3D reconstruction of the hepatobiliary system from in vivo confocal image stacks: relationship of sinusoids to intrahepatic biliary passageways, STII medaka, 12 dpf**. Sinusoids (S) are denoted in red, intrahepatic biliary passageways (IHBPs) in green/gold. All space between sinusoids and surrounding IHBPs is hepatocellular space, not rendered for visual clarity. These types of reconstructions permitted 3D morphometric and volumetric analyses, which assisted in elucidation of hepatobiliary architecture. Grayscale confocal image can be seen in the background. The movie given here, an image capture from an Amira 3D reconstruction, is limited to rotation in a single plane. Actual 3D reconstructions can be rotated in any plane, at virtually any magnification, allowing detailed study of hepatobiliary structure/function relationships. Click here for file

Additional file 8 **Example of 3D projection of liver and gall bladder from in vivo confocal image stacks: STII medaka, 12 dpf**. 3D projections of confocal image stacks, in conjunction with 3D reconstructions, aided in evaluation of 3D architecture of the hepatobiliary system. Intrahepatic biliary passageways (IHBPs) of the liver (L), and gall bladder (GB) were elucidated with fluorescein isothiocyanate. Click here for file

Additional file 9 **Example of 3D projection of parenchyma from in vivo confocal image stacks: STII medaka, 12 dpf**. 3D projections of confocal image stacks, in conjunction with 3D reconstructions, aided in evaluation of 3D hepatobiliary architecture. Intrahepatic biliary passageways (IHBPs) of the liver, denoted by increased fluorescence, elucidated with β-Bodipy C5 Phosphocholine (HPC). Click here for file
